# Graphene enterprise: mapping innovation and business development in a strategic emerging technology

**DOI:** 10.1007/s11051-016-3572-1

**Published:** 2016-09-06

**Authors:** Philip Shapira, Abdullah Gök, Fatemeh Salehi

**Affiliations:** 1 Manchester Institute of Innovation Research, Alliance Manchester Business School, University of Manchester, Oxford Road, Manchester, M13 9PL UK; 2 School of Public Policy, Georgia Institute of Technology, Atlanta, GA 30332-0340 USA

**Keywords:** Graphene, Web content mining, Enterprise development, Commercialization

## Abstract

This paper explores enterprise development and commercialization in the field of graphene. Firm characteristics and relationships, value chain positioning, and factors associated with product entry are examined for a set of 65 graphene-oriented small and medium-sized enterprises located in 16 different countries. As well as secondary sources and bibliometric methods to profile developments in graphene, we use computerized data mining and analytical techniques, including cluster and regression modeling, to identify patterns from publicly available online information on enterprise web sites. We identify groups of graphene small and medium-sized enterprises differentiated by how they are involved with graphene, the materials they target, whether they make equipment, and their orientation toward science and intellectual property. In general, access to finance and the firms’ location are significant factors that are associated with graphene product introductions. We also find that patents and scientific publications are not statistically significant predictors of product development in our sample of graphene enterprises. We further identify a cohort of graphene-oriented firms that are signaling plans to develop intermediate graphene products that should have higher value in the marketplace. Our findings suggest that policy needs to ensure attention to the introduction and scale-up of downstream intermediate and final graphene products and associated financial, intermediary, and market identification support. The paper demonstrates novel data methods that can be combined with existing information for real-time intelligence to understand and map enterprise development and commercialization in a rapidly emerging and growing new technology.

## Introduction

Graphene is an ultra-thin layer of carbon with exceptional properties and the potential for path-breaking applications across a range of areas including strong lightweight materials, next generation electronic devices, specialized coatings, new biomaterials and sensors, and innovative medical applications (Novoselov et al. 2012). Public and private investment in graphene research and innovation has grown in recent years (e.g., BIS 2011; DFG 2011; Moessle and Kurz 2011; Hill 2013; European Commission 2013; Materials Genome Initiative 2014; National Nanotechnology Initiative 2014; University of Manchester 2014; NanoMalaysia 2015). The field is expanding rapidly, with thousands of new patents and numerous companies already entering the graphene domain. However, notwithstanding rising worldwide interest in graphene, there are questions about the positioning of this emerging technology and when promised applications will materialize. This gap between prospective benefits and realized progress is not surprising. Emerging technologies typically face multiple challenges and characteristically go through phases of excitement marked by sharp increases in expectations, interest, and investment, followed by periods of uncertainty and disenchantment. Such cycles may eventually result in the realization of innovation advances and the scale-up of production and use. It is also possible that a vaunted emerging technology either fails to take-off or transitions along pathways not initially expected at the start of the process.

This paper aims to understand and map enterprise development and commercialization in the bourgeoning yet still early stage domain of graphene. In anticipating the prospective development of an emerging technology such as graphene, it is important to examine how the value chain from material inputs to finished products is evolving, and to understand where development is occurring and who is driving it. In particular, it is insightful to go beyond surface-level aggregated trends to probe what is happening at the enterprise micro-level where experimentation and diversity are evident. Tracking and mapping innovation and business development at the enterprise-level, including among small and mid-size enterprises who often seek to pioneer novel applications, is important to those involved in decision-making about graphene research, technology transfer, business investment, and policy. Yet, systematic information about enterprise-level strategies is only partially obtained from conventional public sources. Thus, a second aim of this paper is to examine how new data sources and methods can be employed for real-time intelligence about enterprise development and commercialization of an emerging technology. Using web content mining of the web sites of small and medium-size enterprises in graphene innovation and commercialization, the paper examines application targets, business strategies, and shifts toward specialized applications. Although the paper is focused on graphene, the approaches and methods developed are applicable to other emerging strategic new technologies.

The paper begins with a background review of graphene research and innovation. After explaining the methodological approach and data, we systematically analyze the development and commercialization strategies of 65 graphene small and mid-size enterprises worldwide through web content mining and structured data analysis. Key findings are presented, and there is a concluding discussion of implications and the methodological issues and insights associated with enterprise web mining.

## Background

This section reviews developments in graphene research and innovation. We draw on sources and approaches commonly used in the extant literature, including analyses of scientific publications and patenting, industry and trade reports, and case studies. We consider what these studies tell us about progress and challenges in graphene applications and commercialization.

### Graphene publications and patents

**Fig. 1:**
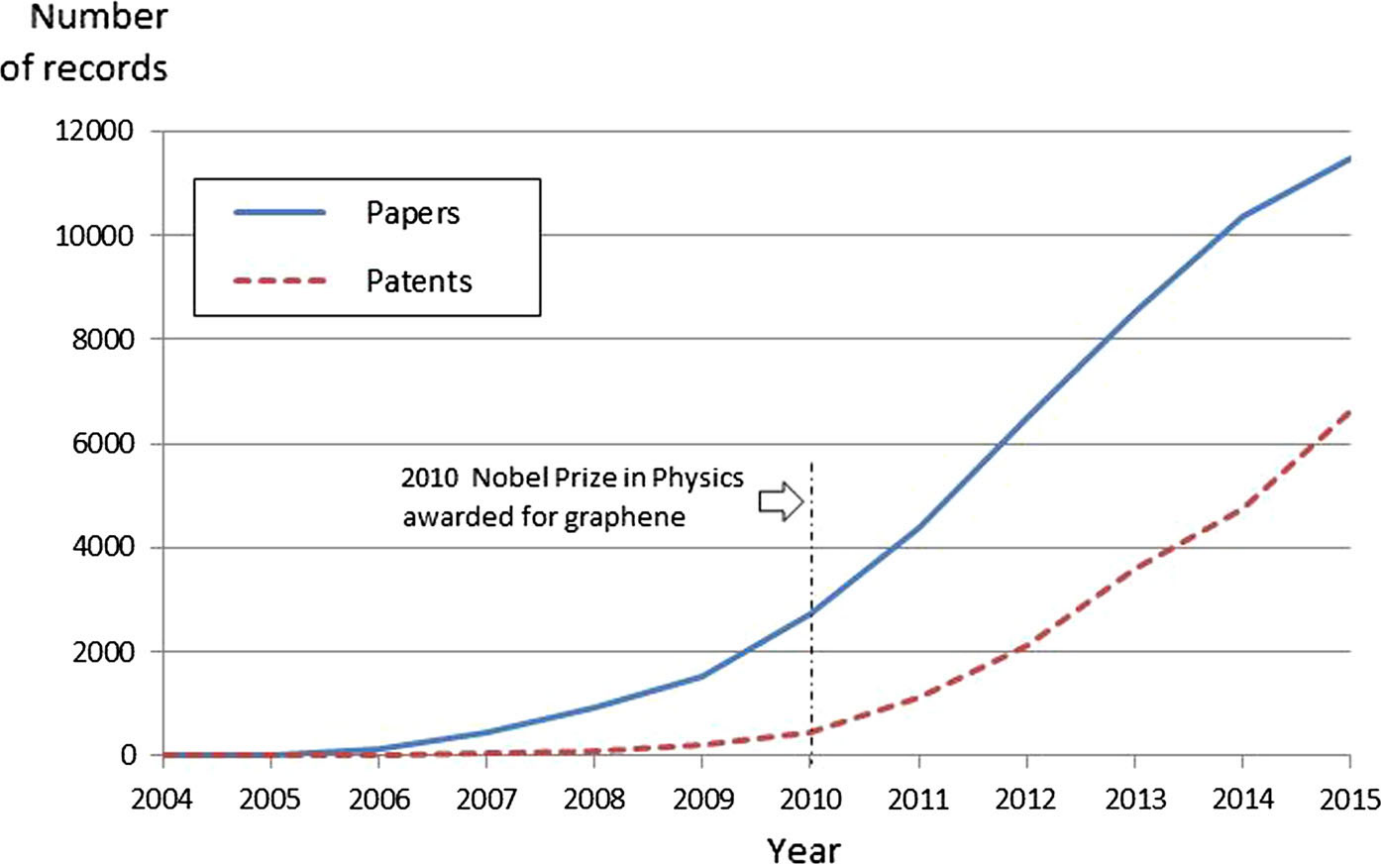
Graphene papers and patents, worldwide, 2004–2015. *Source* (1) Analysis of scientific papers (articles, proceedings papers, and reviews) with “graphene” in title, Web of Science, publication years 2004–2015 (*N* = 47,074); and (2) analysis of patent applications identified by “graphene” in title or topic fields, 2004–2015 (*N* = 19,0402), Derwent Innovations Index, Thomson Reuters

Bibliometric data on scientific publications are valuable in investigating patterns in graphene publications, citations, and research collaborations. Our own analysis of Web of Science data shows a rapid overall growth of graphene research and resulting scientific publications over the last decade (Fig. 1). Between 2001 and 2004, there were fewer than 40 scientific papers on graphene published worldwide. In 2007, there were about 450 papers, increasing to over 4350 in 2011—the year following the 2010 Nobel Prize in Physics award for earlier pioneering work on graphene (Royal Swedish Academy of Sciences, 2010). Publication growth has continued, with some 11,500 graphene papers published worldwide in 2015. Researchers based in China are now the largest producers of graphene publications, overtaking the USA in 2011.

Bibliometric analysis also helps to distinguish emergent graphene science topics. Small et al. (2014), using Scopus data (1996–2010), identify emerging topics in graphene to include graphene nanosheets and nanocomposites, epitaxial graphene, nanoelectronics, nanoribbons, quantum transport, and mechanical properties of graphene. Chen et al. (2013) spotlight scientific knowledge diffusion paths of graphene for optoelectronics. Using citations in Web of Science data (2004–2012), they identify key subareas of graphene research for optoelectronics including reduced graphene oxide methods, chemical vapor deposition, and exfoliation techniques. Gomila and Marro (2013) combine tech-mining with semantic-TRIZ (Theory of Inventive Problem Solving) to highlight an increasing trend in the use of graphene in cathode materials, mainly to enhance the conductivity and the discharge and recharge of lithium-air batteries.

A rapid worldwide growth in graphene patenting activity is also evident. Graphene patenting took off about 2011 (when there were thirteen times more applications published than three years earlier in 2008). In 2015, more than 4700 graphene patent applications were filed worldwide, based on our analysis of Derwent Innovations data (Fig. 1). Early corporate entry and activity in graphene has been analyzed by using evidence from corporate publications and patents across country and application lines (Shapira et al. 2012). This research suggests that corporate inventive activities in graphene are occurring in a concurrent pattern with developments in research. Enterprises are currently interested both in graphene exploration and in its exploitation.

The rise of Asian industrial interest in graphene, not just in the Japan and South Korea but also in China, has attracted attention. Baglieri et al. (2014) investigate patenting in carbon nanotubes and graphene in Japan and China, finding differences in the type, fragmentation, and concentration of patent ownership. They show a dispersed and industry-oriented model in Japan. The largest Japanese patent holders in graphene are mainly large electronic companies (such as Toshiba, Sony, Sekisui Chemical, Fujitsu, Hitachi, and NEC), specialized R&D companies (e.g., Semiconductor Energy Laboratory Co. Ltd.–or SEL), and public laboratories (such as the National Institute for Materials Science). In contrast, in China, a university-oriented model is commonly found, although more recently several Chinese companies have become prominent in graphene patent applications.

Work published by the UK Intellectual Property Office indicates an accumulation of over 13,000 patent family applications relating to graphene by 2014 (IPO 2015). Academic applicants seem to be interested in a broader range of potential graphene applications, reflecting their exploratory mission. The top corporate patentees have a narrower technology focus than top academic institutions, with Samsung being an exception (IPO 2013). In the UK, there is a mix of academic and corporate patenting, as in other countries, but no British-based company has developed a large graphene portfolio (IPO 2015). When normalized, the UK effort in graphene patenting looks weaker compared with several other leading countries and the UK’s own scientific power in graphene.

### Graphene production and market trends

The absence of cost-effective and scalable graphene manufacturing techniques is a major current challenge. Ideal large-sized mono-crystalline single-layer sheets of graphene are hard to isolate and costly to produce, while much of the graphene now producible has inferior performance levels (IDTechEx 2012). Available manufacturing techniques include variations on the original scotch-tape method and several more sophisticated techniques including chemical vapor deposition (CVD), liquid-phase exfoliation, plasma, and oxidization-reduction (Warner et al. 2013). CVD and exfoliation have been the two most commonly used methods (Sivudu and Mahajan 2012).

There is an inverse relationship between scalability of these techniques and their costs, quality, and range of accessible applications (IDTechEx 2012). At present, most companies are relatively low-volume graphene materials producers, and few are moving down the value chain to offer intermediate products (such as functional graphene inks) and graphene-enabled devices. The downstream graphene value chain offers the promise of higher returns, as upstream materials usually represent only a part of the costs of a downstream product, and there is greater potential to differentiate downstream products so as to capture price premiums for specialized functionalities. However, moving along the value chain is not straightforward, as it requires skills that are different from graphene production. The expansion in patents is also a potential intellectual property issue to be navigated by firms involved in graphene intermediates and device production. IDTechEx’s (2012) market forecast anticipated additional near-term potential markets (by year 2018) for graphene materials in super-capacitors, high-strength composite materials, touch screen (transparent) conductors, radio-frequency identification (RFID) devices, smart packaging, and sensors. Their 2014 market forecast expects the market for graphene material to grow from around $20 million in 2014 to more than $390 million in 2024 (IDTechEx 2014). The end value of the final products enabled by graphene is anticipated to be much higher (Graphenea 2014). Currently, there is demand from research institutions for small quantities of high-quality graphene. Future Markets (2015) finds that market demand for graphene is currently relatively small and there is an over-supply of graphene, especially for low-quality graphene material.

At present, the number of companies involved not just in research but also in the manufacturing and marketing of graphene and graphene-enabled products is relatively small, although growing. Among early studies, IDTechEx (2012) reported on the graphene-related activities of 32 companies (both large and small), while Future Markets identified 18 companies and universities active in the field of graphene in their 2012 report (Future Markets Inc 2012). IDTechEx (2012) indicated that large companies, as well as venture capital funders, were investing in selected small graphene companies. Mostly, these companies were in the pre-growth stage. More recent studies report growth in the number of firms engaged in graphene-related activities. Graphene Tracker (2015) reports more than 60 companies worldwide involved in graphene-related activities, of whom over 30 are engaged in producing graphene, nearly 20 in supplying graphene materials, 6 in making CVD or characterization equipment, and about a dozen in making advanced graphene components and end products. A 2015 study suggests that over 200 companies are now making graphene or developing graphene applications (Future Markets Inc., 2015). This estimate includes large firms as well as small and medium-sized enterprises (SMEs).

### Graphene value chain, regulation, and commercialization obstacles

From a commercial perspective, graphene generates value when it is input or embodied into products, processes, devices, and systems that can be marketed to users. Wei and Kivioja (2013) identify three major stages in the graphene value chain. The first stage involves graphite ore supply and the development of specialized manufacturing machines—for the production of graphene material, for example, by solution-based methods (liquid exfoliation from graphite ore) or CVD methods. The second stage involves graphene-derived materials production and supply, with the third stage involving graphene devices and products. Wei and Kivioja observe a range of companies engaged in and along these stages of the graphene value chain, with academic researchers also engaged in exploring new approaches. There are variations in the interests of those involved in the graphene value chain. For example, SanDisk exhibits specific interests in memory device applications. Similarly, larger corporations, such as IBM, Xerox, McAlister Technologies, and Bayer, have focused interests, as do small dedicated firms. Samsung appears to have the broadest technological interests in graphene.

Firms that currently produce and supply graphene directly to the open market are generally small to medium-sized. There are now many available formats of graphene including graphene films, layered materials, composites, and powders with variations in scale, purity, and performance characteristics. Graphene can presently be purchased in low volumes and at relatively high prices. Several companies, such as US-based Graphene Laboratories Inc., sell graphene products online (typically targeted to research laboratory needs). It is anticipated that graphene prices will decrease as industrial-scale-up occurs, although as yet graphene priced on the market appears not to have entered a significant downward price curve. Over the longer run, it continues to be expected that the development of new technologies will facilitate large-scale production and lower costs—and there have been numerous recent media announcements of novel methods that promise cost-effective graphene mass production (for a sample of such announcements, see Saltarin 2014; ITV 2014; Wenz 2015).

The performance-price relationship is an important factor in graphene’s development. Some users are willing to pay high prices for superior performance, for example in specific military applications or for very high-speed computing. More typically, potential users will compare the performance-price relationships offered by graphene with those of the materials in current use. In many cases, for example in the use of silicon in transistors, there are incumbent materials that are widely used and which can be manufactured cost-effectively at scale. Although advances in graphene manufacturing promise scale-up and price-reduction opportunities, existing materials are also subject to technological and manufacturing improvements. Alcalde et al. (2013) assess the competitive advantage of graphene over incumbent materials in a number of products and applications. They note the steep increase in graphene patents compared to other materials (e.g., carbon nanotubes and carbon fiber) and the wide variety of application areas for graphene (i.e., its generic nature) as two indicators for accelerated commercial activity in this area. Yet, the unique properties of graphene might not be sufficient for its commercial success. Three main issues are highlighted that will likely influence the industrial uptake of graphene: (1) cost competitiveness, scalability, and reliability of graphene manufacturing; (2) its suitability for application in industrial production methods (i.e., the technological and industrial readiness of value chains that would take up graphene compared to standing alternatives; and (3) sociopolitical considerations, legislation, and industrial development policies (Alcalde et al. 2013).

## Applying web mining to map graphene enterprise strategies: methodology

The preceding review of a series of existing studies provides a broad picture of graphene research, patenting, manufacturing, and early commercialization. It also raises many questions about the strategies of companies engaged in graphene. In this part of the paper, we explore the application of enterprise web mining to graphene companies. We seek to go beyond what is available from conventional publication and patent sources, and to probe at a more disaggregated enterprise-level than in available secondary reports. We draw on research that is developing new and scalable methods to mine and combine information from unstructured online sources including enterprise web pages. We focus on a worldwide set of SMEs that are engaged in graphene. While concentrating on SMEs presents limitations, we posit that the strategies of smaller firms may be especially insightful in understanding discontinuous and more disruptive approaches to innovation (see also Akerman 1999). The next section of the paper explains web mining and its uses and limitations in enterprise analysis. We then present results from our web content analysis of a worldwide sample of 65 graphene SMEs. Using information extracted from the web sites of these companies, we profile graphene activities and firm characteristics, applications and products, value chain positions, clustering, and factors influencing graphene product introductions.

### Uses of web mining in analyzing enterprise development

Web mining uses computerized data mining methods and analytical techniques to discern and extract patterns from publicly available online web sites and pages (Liu 2011). A particular application area for web mining is in the study of enterprise strategy and innovation (see Youtie et al. 2012; Gök et al. 2015). There are both advantages and limitations in using websites as a source of information about business enterprise. Website information has the benefit of being publicly available. It is most readily analyzed for SMEs, where sites are more focused, rather than large multi-divisional transnational corporations. It has been observed that SME websites often present strategic-oriented text about products, partners, and customers, while the websites of large companies are frequently oriented to public audiences (Li et al. 2016). The great majority of technology-oriented SMEs have a web site presence. Different languages are used—although it is common to find English or an English version (and machine translation can be applied). Information on corporate web pages is, of course, self-reported by those companies: there is no standard format, and there are differences in the type and amount of information disclosed. Many companies present detailed information on their products and technologies as well as information on their history, managers, business locations, facilities, and other news items. Other companies are much sparser in what they present. Companies naturally seek to promote themselves. While there is no formal validation of information presented on websites, since companies are in business and need to maintain the confidence of customers, suppliers, and investors, there is an incentive not to mislead in terms of what is presented. At the same time, companies are not expected to post information that is confidential, proprietary, or critical.

In many (although not all) cases, older versions of web pages can be found through use of internet archives such as the Wayback Machine (http://archive.org/web/), allowing the building up of a data series of developments over time (Arora et al. 2015). Web sites are accessible for companies in countries around the world. In some countries, for example in China, where there are few if any freely accessible structured databases of corporate business information, analyzing online information presented by firms is a useful unobtrusive data collection approach particularly for smaller firms. Additionally, while conventional structured databases, including databases on publications and patents, make information available about the early stages of research and development, web mining can capture information about corporate innovation activities that are more downstream. Studies that have matched enterprise web-mined data with information obtained from other information sources find broad validity and usability (Li et al. 2016; Rietsch et al. 2016). Moreover, one recent study indicates that UK manufacturers in green goods sectors are far more likely to report various kinds of research, development and innovation activities (including scientific research, technology development, and product development) on their web sites than what might be evident from analyses of databases of patents, publications, public R&D grants, and financial reporting of R&D (Gök et al. 2015). On the other hand, there can be periods when technology companies “go dark” and neither update on everything they are working on nor provide great technical detail since this would then become public knowledge available to competitors. Corporate websites vary greatly in their underlying technologies (e.g., dynamic websites versus static websites), depth (number of pages they contain), and purpose (websites providing information versus online shopping outlets). Some firms have websites in multiple domain names (e.g., separate domains for different brands), while others share a website with other firms (e.g., parent company has a website, while subsidiaries share the same domain). Some firms present information in multiple languages, while others only have English versions. Overall, the greatest challenge of web content mining at scale is that of managing and sorting through a great deal and variety of material: processing and analyzing that information can be complex and difficult to manage through reasons of its global scope, sheer size, and unstructured nature.

While recognizing the limitations of web mining, the increasing amounts of information now being made available online by companies present opportunities to explore what can be gained from this source. Although there are challenges, new methods of large-scale data analysis can be helpful in processing and analyzing the data to discover and discern useful insights. There is already some evidence on this point. In earlier research involving one of this study’s authors, web mining methods were piloted to investigate commercialization strategies of twenty graphene SMEs in three countries, namely the USA, UK, and China (Arora et al. 2013). This analysis classified graphene SMEs into three groups: specialized product development firms, specialized material development companies, and firms with integration into existing product portfolios. Graphene SMEs with specialized products tend to focus on applications and end-users, have greater reliance on university links, and exhibit born global characteristics. Companies with a specialized material focus are mainly science-based firms, also reliant on relationships with universities but with a local orientation. Graphene SMEs that integrate graphene development with their existing portfolio of nanotechnology products exhibit diversified R&D activities and are less associated with universities. The comparison by countries showed that the UK graphene SMEs tend to produce graphene for basic and applied research and are more science-driven and less application-oriented. However, USA and Chinese SMEs are more connected with applications and demonstrate greater engagement in commercialization processes with the potential growth prospects (and risks) associated with this orientation. An interpretation of this result is that in-country clusters of downstream developers, manufacturers and assemblers in electronics, telecommunications, and other consumer technologies in the USA and China raise proximate market demand prospects for scaled-up graphene applications that are not so strongly present in the UK.

The world of graphene has substantially expanded since 2011, when this earlier study first collated its web-mined data. There have been advances in research, great growth in patenting, more attention to moving graphene out of the laboratory and into industry, and growth in public initiatives to foster innovation in graphene. New companies have been formed in multiple countries, a greater range of companies now manufacture and supply graphene, and graphene products are now appearing on the market. While there continues to be much optimism about graphene’s potential, there is also a greater realization of the complexities of commercializing a fundamental innovation in materials (Colapinto 2014). Nonetheless, further investments are being made in graphene development and applications. In sum, it is a timely moment to apply web mining to explore the development and technological and business strategies of today’s innovative graphene companies—focusing on the larger set of graphene SMEs that are now in operation around the world and using available enhancements in methodological techniques.

### Our approach to enterprise web mining

For the present study, we developed an approach to extract information from enterprise websites, focusing on graphene companies. This extracted information is analyzed to discern the characteristics and strategies of the firm and the nature of its graphene commercialization strategies. A core methodological challenge in this process is that information contained in corporate websites has no standard schema or format (i.e., the data are unstructured)—whereas analysis is most readily done when these data are transformed into a structured format. In this study, the process of converting unstructured data into a structured format that can be analyzed involves web content retrieval, text mining, and statistical analysis. We used and developed a methodology based on Gök et al. (2015) and trialed several different software packages. We finally used this combination: IBM Watson Explorer (a software package available under an academic no-cost license that we used to search and index enterprise website data); VantagePoint (a licensed text mining and analysis software package used to clean data and extract variables); and Stata and SPSS (standard statistical analysis software packages).

We included graphene-based SMEs in our database and excluded firms whose primary activity was not related to graphene. We also did not focus on large multinational companies. We used a mixed-methods process to identify companies that could potentially be included in our data set of graphene SMEs. We started by identifying the assignees of graphene-related patents. This search revealed a number of larger corporations that were active in graphene patenting. Some smaller firms with graphene patents were also identified. This was helpful, but this approach clearly did not cover all enterprise activities in graphene because many SMEs do not patent (or have yet to patent). Therefore, we also used other sources to identify these firms including social media, specialist websites, academic publications, business databases, industry reports, and also gray literature. Our initial worldwide data set consisted of 87 graphene-based SMEs.

The process of web content mining started with crawling the websites of these graphene-based SMEs. Web crawling (also known as web scraping) is a computerized method to harvest and extract information from web sites and web pages. We used IBM Watson Explorer for this process. The web crawling process for the data used in this study was initiated in September 2014 and updated in January 2015. Our review during this crawling process led us to focus on 65 firms (74 % of the initial sample). We excluded firms that on closer investigation were really focused on consulting services rather than graphene-related manufacturing. Other firms that dropped out were ones that appeared to no longer be in business at the time of crawling or for whom little useful information could be obtained from their web sites. The final data set does comprise graphene SMEs who are in the business of advancing and developing graphene technologies. As we show later, while there are differences in detailed characteristics, most of these graphene SMEs are upstream in the value chain, making graphene materials, some intermediate products, and equipment for graphene manufacturing. The data set by definition does not include SMEs who do not publicly present online what they are doing in graphene. The crawling process targeted web addresses or, more specifically, Uniform Resource Locators (URLs). In total, some 74,038 URLs were initially crawled. After deleting duplicates, the number of unique URLs associated with the data set was 67,672. There are variations among the firms: some enterprise web sites comprised a few web pages, while others had very large websites in excess of one thousand web pages.

After IBM Watson Explorer finished crawling, we indexed the corresponding corpus by using the same tool. This indexing process discarded web pages that did not contain any valuable information. We indexed 11,285 pages. Most of these pages (about 70 %) were in standard HyperText Markup Language (HTML) format. The data set also included text extracted from files in Portable Document Format (PDF), plain text, Extensible Markup Language (XML), Microsoft PowerPoint, and other file types. The resulting corpus comprised more than 9.45 million words. The indexing process included the following four components: (1) language detection, which removes non-English text not filtered out in the crawling process; (2) URL rule-based annotation, where sections of the corpus are tagged by firm names based on extensive rules; (3) linguistic analysis—the corpus is divided into paragraphs and sentences, phrases and words. Each word is stripped of its stemming (e.g., “manufacturing” or “manufactured” is converted into the root form of the word, “manufacture”). Words are tagged by their part of speech (i.e., noun, adjective, and verb); and (4) named entity recognition annotations: locations, person names, and organizations are tagged in the text by using an advanced UIMA (unstructured information management architecture)-based algorithm. We utilized IBM Watson Explorer Studio in creating some of the UIMA based rules.

We exported the indexed corpus into XML and CSV (Comma-Separated Value) files so that it could be imported into VantagePoint software. We also created a conceptual map of the variables we sought to extract from the data by using a variety of sources including technical academic and non-academic publications, consultations with graphene experts, and other studies of graphene. A list of the variables and associated extraction rules we used is presented in Appendix Table 4. A common feature for all the variables constructed is that we conducted extensive data cleaning. We designed our construction rules to minimize noise and error. However, due to the nature of our corpus, initially some false positives were captured in almost every variable. We investigated every variable and excluded irrelevant captures. After we constructed our variables in VantagePoint software, we created a two-dimensional structured table consisting of firms in row and variables in columns.

The final stage of our method involves statistical analysis. We imported the structured table created by VantagePoint into Stata and SPPS. We further processed the data by creating new variables and modifying the existing ones for statistical and cluster analysis. In the statistical analysis, we introduce a normalization process. The websites varied greatly in terms of web pages (range of 1–2459, with a mean of 162) and words (range of 10–4,435,751, with a mean of 136,963). We thus normalized all variables by the total number of words they contained then multiplied by a factor of 1000 for ease of comparison.

Our validated data set comprised 65 small and medium-sized graphene-based firms. Identifying SMEs is inherently a moving target. The number of graphene companies has increased over the past few years, but there is also underlying churn as new firms are established, some are taken over by other companies and merged, while others may go out of business. Our validation process excludes wholesalers and larger firms. In some countries, particularly China, we have not detected all SMEs, and this underrepresentation should be kept in mind when comparing across the regions. In the other regional locations (North America, the UK, and Western Europe), our coverage is better. Overall, we do have representation of SMEs from the world’s major regional locations for graphene research and innovation and, based on our available knowledge, the data set represents the largest set of graphene SMEs subject to web mining to date.

### The graphene companies

The 65 small and medium-sized graphene-based firms in our data set are based in 16 different countries. We constructed four regional groupings of firms for further analysis. The North America group constitutes around half of the data set and comprises 32 USA and two Canadian firms. A second group (16 % of the data set) is comprised of ten UK firms. The third group (19 % of the data set) comprises 12 firms located elsewhere in Western Europe, of which six are based in Spain, two in Norway, and one each in Germany, Italy, the Netherlands, and Sweden. The fourth group (17 % of the data set) is comprised of 11 companies located in East Asia and emerging economies, of which three are based in China, two in South Korea, and one each in India, Japan, Malaysia, Russia, Thailand, and Turkey. There is some logic in clustering these countries together in that the varieties of economic management in the countries of the East Asia and emerging economies group emphasize government industrial targeting and control that is more intense than typically found in Western European and North American economies.

**Fig. 2:**
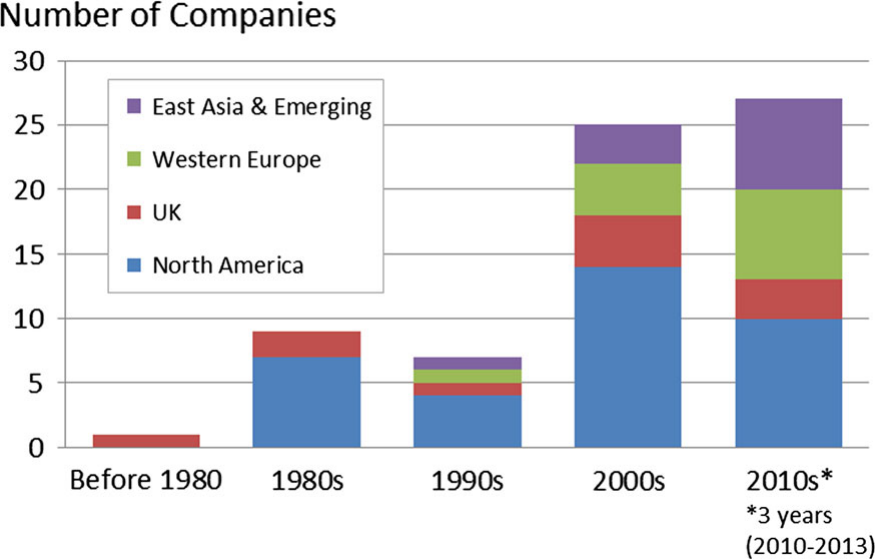
Period of founding, graphene SMEs, by region. *Source* Web mining analysis of 65 graphene small and medium-sized enterprises (SMEs) in study data set. See *text* for additional details

About three-quarters of the 65 graphene SMEs in our data set were established after 2000 (Fig. 2). Companies founded since 2010 (27 firms) comprise the largest segment, including 13 firms established since 2012. We also include firms founded prior to 2000 that have since changed their focus to graphene. Firms located in North America and the UK are prominent among older firms as well as those established in the 2000s and 2010s. Firms based in Western Europe and in East Asia and emerging economies are notably prominent among firms founded in the 2010s (note that this period comprises 2010–2013).

## Findings

### Graphene activities and firm characteristics

In analyzing the web sites of the data set of our graphene SMEs, we probe several key characteristics. These include the extent to which the firms emphasize graphene in their text (graphene intensity), the extent to which R&D activities are mentioned, the properties of graphene highlighted, graphene production methods, and mentions of manufacturing and service activities.

**Fig. 3:**
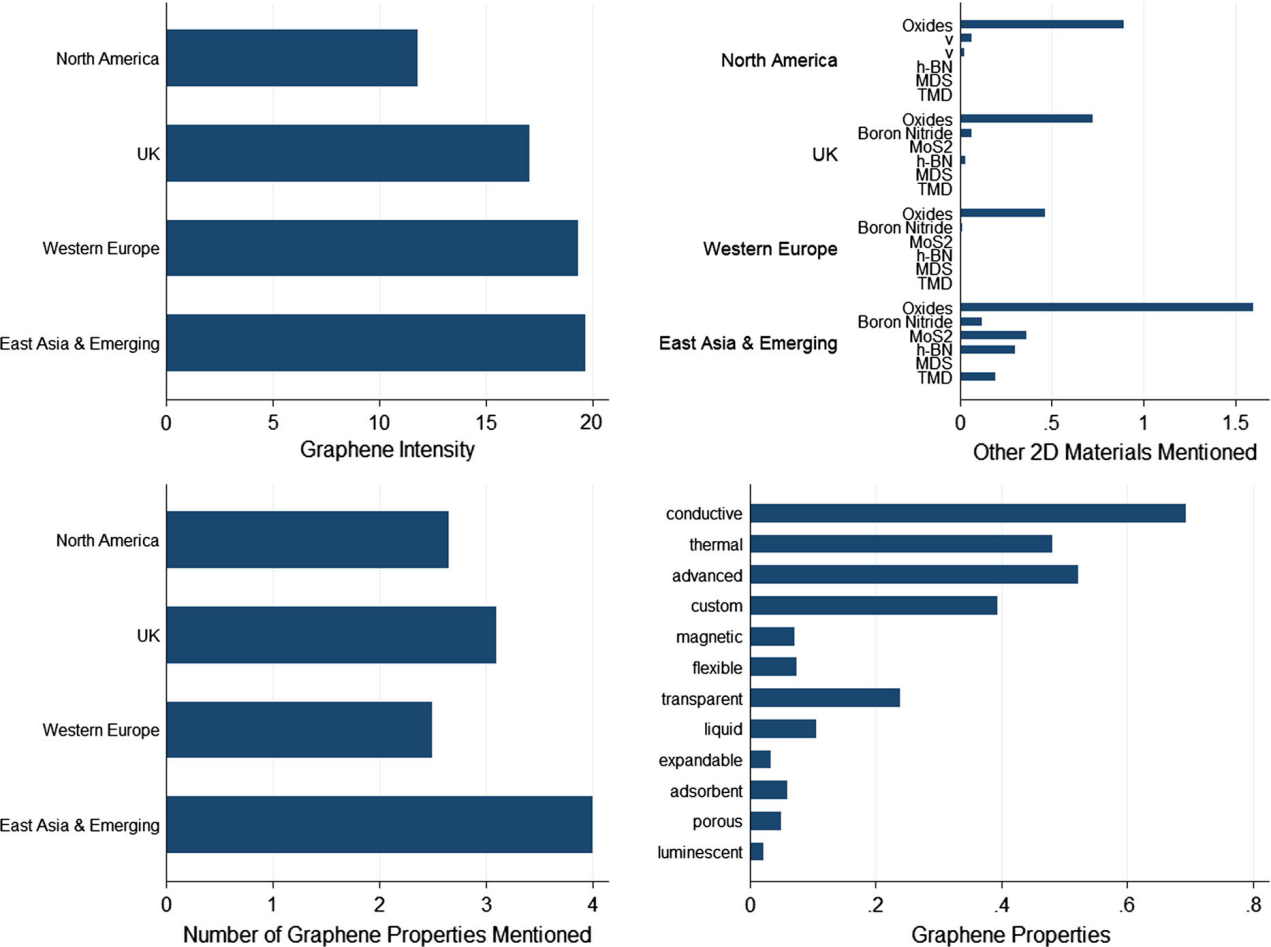
Graphene intensity, graphene properties, and other 2D materials. *Source* Web mining analysis of 65 graphene small and medium-sized enterprises (SMEs) in study data set. *X*-axis is normalized scale per 1000 words. *Top left* diagram shows the relative intensity of mention of graphene (based on normalized mentions per 1000 words). *Top right* diagram shows the mention other two-dimensional (2D) materials (based on normalized mentions per 1000 words).* Bottom left* diagram shows the average number of mentions of graphene properties. *Bottom right* diagram shows the average number of graphene properties mentioned. See *text* for additional details

As would be expected from the sample selection, the firms in our data set make many references to graphene. Relatively (based on normalized mentions per 1000 words), graphene intensity is lower for US-based firms, while it is higher for firms based in Western Europe and in East Asia and Emerging countries (Fig. 3, top left). Higher graphene intensities might indicate that firms are focused on graphene itself as a material commodity. Additionally, the firms in the sample cite a wide variety of properties of graphene. The most common properties are conductivity, thermal, transparency, flexibility, and liquidity, while a range of other properties such as magneticity, expandability, adsorbent features, luminosity, and porosity are also raised (Fig. 3, bottom right). Firms mention on average two to three properties of graphene in their website, while this number increases to around four for firms based in East Asia and emerging economies (Fig. 3, bottom right). Firms also mentioned other two-dimensional materials, particularly oxide-based materials. Firms from East Asia and emerging economies tend to more often reference other two-dimensional (2D) materials and they mention a wider variety of these other materials (Fig. 3, top right). Mentioning more properties of graphene and also discussing other 2D materials may indicate that firms have a broader and more exploratory technological scope.

**Fig. 4:**
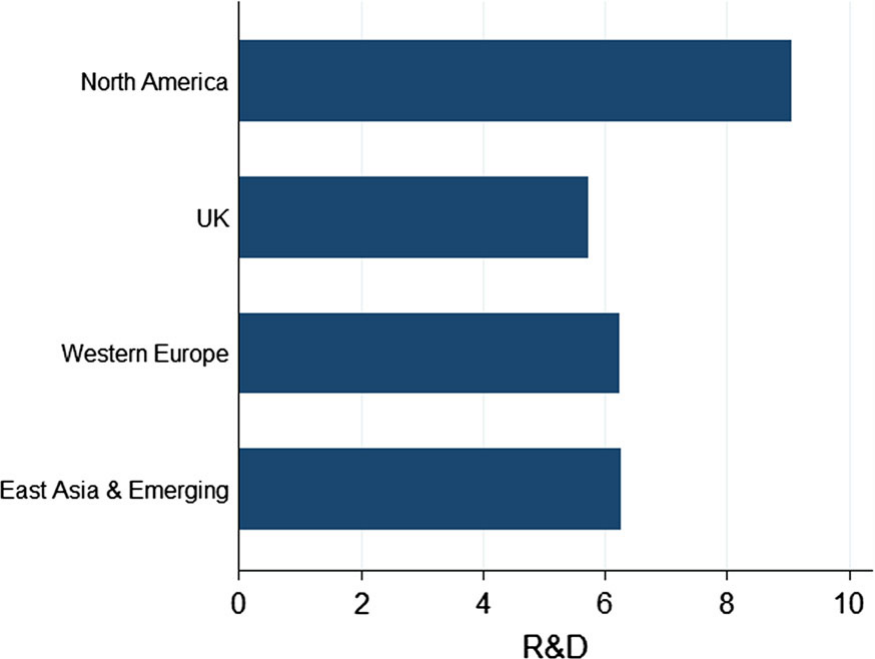
R&D mentions by graphene SMEs. *Source* Web mining analysis of 65 graphene small and medium-sized enterprises (SMEs) in study data set. *X*-axis is the mention of R&D activities normalized scale per 1000 words

In general, the firms are R&D intensive. However, only five firms produced graphene-related scientific publications, while 18 firms have graphene patents. In total, only 21 firms (around 30 %) have either patented or published. This highlights the point that if we selected firms based only on conventional publication and patent data, we would miss around two-thirds of the graphene firms included in our web content mining sample. Nonetheless, graphene-based SMEs depend on science and technology, and they frequently mention other research and development activities in addition to papers and publications. Firms from North America tend to mention research activities relatively more, while UK firms tend to mention R&D relatively less frequently (Fig. 4). Among the companies in our sample set, CVD techniques for making graphene are most commonly mentioned. However, firms also mention other techniques such as exfoliation, intercalation, epitaxy, spin coating, and molecular assembly.

We have extracted the applications of graphene mention on the websites of the SMEs included in our sample (Table 1). Current applications of graphene range from graphene-based paints to graphene ink to capacitors and other devices used for energy storage. Similarly, firms mention a wide range of potential applications of graphene under development such as anti-corrosive coatings used in electronics and electrical equipment, photovoltaic devices for solar cells, polymer composites for dental care, and advanced graphene-hybrid admixtures.

**Table 1:** Offered and potential final products. *Source* Web mining analysis of 65 graphene small and medium-sized enterprises (SMEs) in study data set

Offered final and intermediate products	Potential final and intermediate products
Graphene field effect transistors	Anti-corrosive coatings used in electronics and Electrical equipment/photovoltaic devices for solar cells/polymer composites for dental care
Thin film transistors (TFT)	Ultrafast photodetector
Graphene field effect transistors	Nanocomposites
Graphene-based paint	Advanced graphene-hybrid admixtures
Functionalized graphene, inks, and coatings	Graphene ink
Graphene ink	Solid-state nanopore sensing platforms
Ultracapacitors/energy storage	Electrodes for super capacitors and batteries
Ink and coatings for the printed electronics	Composite of silicon and graphene for longer lasting, faster charging batteries
	Energy storage materials, inks, and coatings
	Composites and film adhesives

**Fig. 5:**
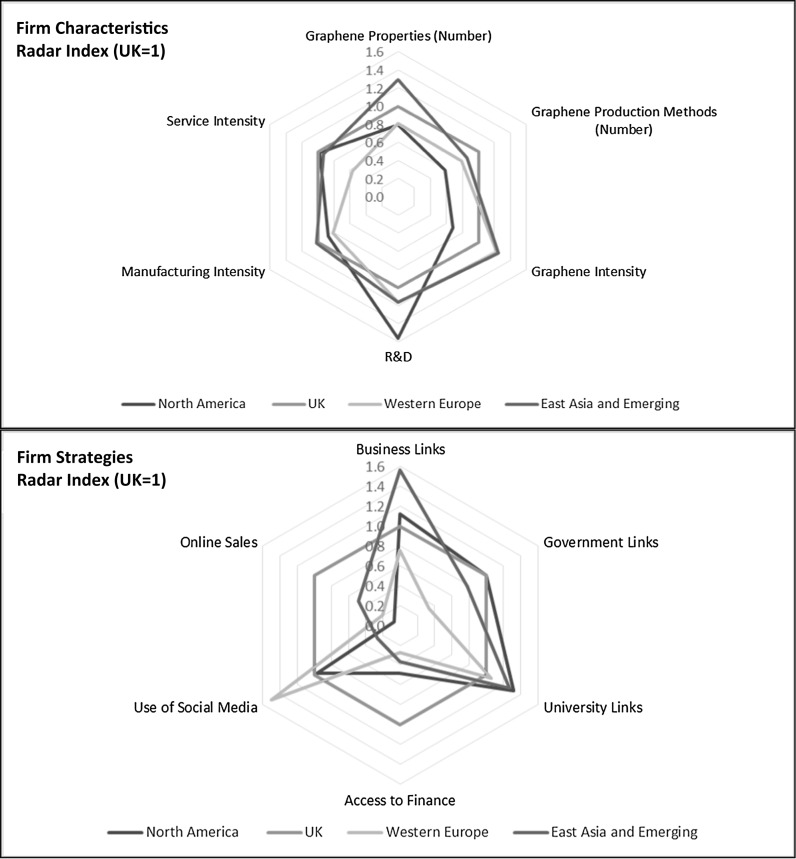
Graphene SME characteristics and strategies—comparative analysis by regions. *Source* Web mining analysis of 65 graphene small and medium-sized enterprises (SMEs) in study data set. Normalized to UK = 1. The *top radar* diagram plots the values for mentions of R&D, graphene intensity, service and manufacturing intensity, number of graphene production methods, and number of graphene properties for each region. The *bottom radar* diagram plots the values for mentions of business relationships, links to governments and universities, access to finance, online sales, and use of social media for the four major regions. See *text* for additional details

A summary comparative analysis highlights the key similarities and differences of the characteristics of the firms by the four major regions (Fig. 5, top). In this analysis, the UK is normalized to a factor of one. Compared to UK firms, North American (mainly US) SMEs involved in graphene report that they give greater stress to R&D activities on their web sites. As discussed earlier, this is measured by mention of R&D-related keywords normalized by number of words in web sites, although we do not have contextual information as to how exactly these words are used. Additionally, lower mentions of graphene itself relative to other topics suggest that at least some North American firms are focused further along the graphene value chain, highlighting the features of their products rather than of graphene. Our further analysis of value chain position (discussed later) adds weight to this proposition, showing that more than half of the North American graphene firms in our sample target intermediate products or equipment making. Compared to the UK firms, Western European and East Asian and Emerging Economy graphene SMEs give greater weight to mentioning graphene and are slightly more likely to emphasize R&D activities on their web sites. East Asian and Emerging Economy graphene SMEs emphasize a wider range of graphene properties. Most of the firms highlight graphene manufacturing activities, although some also mention service offers (such as consultancy). While this is broadly the case for all regions, UK and East Asia and Emerging Economy firms are slightly more manufacturing and slightly less service oriented.

### Firm strategies

Relationships with other firms, universities, and government are strategically important to the development of high-technology SMEs. Similarly, access to private sector finance is also strategically important for development and growth of SMEs. Our analysis of graphene SME enterprise web sites allow us to discern what these companies present on these vital relationships. We do find that there is frequent mention of linkages with other businesses, universities, and government. In general, mention of linkages with businesses and universities is more common. There are also regional variations in linkages. Firms from East Asia and emerging economies more frequently report business links than firms elsewhere, suggesting that they are part of broader business clusters and alliances. UK and Western European graphene SMEs are relatively less likely to mention other external business relationships. Western European SMEs cite government linkages relatively less frequently. The UK graphene SMEs tend to mention university linkages less frequently than firms elsewhere. North American and UK graphene firms are more likely to mention government linkages. Interestingly, UK firms are more likely to mention linkages with external sources of private finance, including venture capital and equity capital. Other Western European firms are relatively less likely to mention external private sector finance sources (Fig. 5, bottom).

We have also examined online ways in which companies market and communicate. Firms in the UK and, to some extent, in East Asia and emerging economies are more frequently likely to report an online sales capability on their web site. Marketing through direct online sales may suggests that firms are more likely to be focused on low-volume commodity graphene materials for research laboratories. It could also suggest capability and trust in online sales processes. We find that many graphene-based enterprises use social media to provide information on their activities and products. An exception is that firms based in East Asia and emerging economies are less likely to use social media. Compared with the UK firms, graphene SMEs in East Asia and emerging economies are more likely to highlight relationships with other businesses, but less likely to emphasize external sources of private finance. These companies may be able to raise funding internally, informally, or from other business partnerships—and they are relatively less willing than UK companies to report their sources of finance on their web sites. Graphene companies in all four regions report relationships with universities, but the UK graphene companies are slightly less likely to stress such relationships than companies elsewhere. North American and UK companies are more likely to report government relationships on their web sites. UK companies are more likely to report online sales capabilities. As a side note, we found that graphene firms based in the UK and East Asia and emerging economies are more likely to mention the graphene Nobel Prize in Physics than firms based elsewhere (Fig. 5).

### Graphene value chain positions

Value chain analysis is a tool for systematically examining the positioning of firms and identifying sources of competitive advantage (Porter 1985). It has also been used as a broader concept to analyze the larger stream of activities, “the value system”, in which a firm’s value chain is embedded (Porter 2001: 50).

We use a value chain position typology developed by Lux Research (2007) originally for nanotechnology. This classification has been used in subsequent research analyzing nanotechnology-based corporate activity (Wang and Guan 2012; Alencar et al. 2007). The typology comprises “nanomaterials” (nanoscale structures in unprocessed form, e.g., nanoparticles, fullerenes, graphene powder), “nanointermediates” (products with nanoscale features such as nanocomposites, coatings, and fabrics), “nanotools” (equipment used to visualize, manipulate, and model matter at the nanoscale) and finally “nano-enabled products” (finished goods incorporating nanotechnology). Nanomaterials constitute the upstream part, nanointermediates are in the midstream, nano-enabled products are in the downstream, and nanotools are spanned along all the stages of the value chain from upstream to downstream. Drawing on this typology, we devised four categories of graphene value chain positions for our sample of 65 graphene SMEs:

- *Material producers* firms producing nanoscale graphene-based structures in unprocessed form, e.g., graphene powder, nanoplatelets, dispersion, graphene oxide
- *Intermediate producers* firms producing products with nanoscale features that are further incorporated into other products, e.g., graphene-based composites, coatings, inks, battery additives, transistors
- *Equipment manufacturers* firm producing tools and equipment used to visualize, manipulate, and model matter at the nanoscale, e.g., CVD graphene-producing machine
- *Final product manufacturers* firms producing finished goods incorporating graphene, e.g., solar cell, paints, DNA sequencing devices

**Fig. 6:**
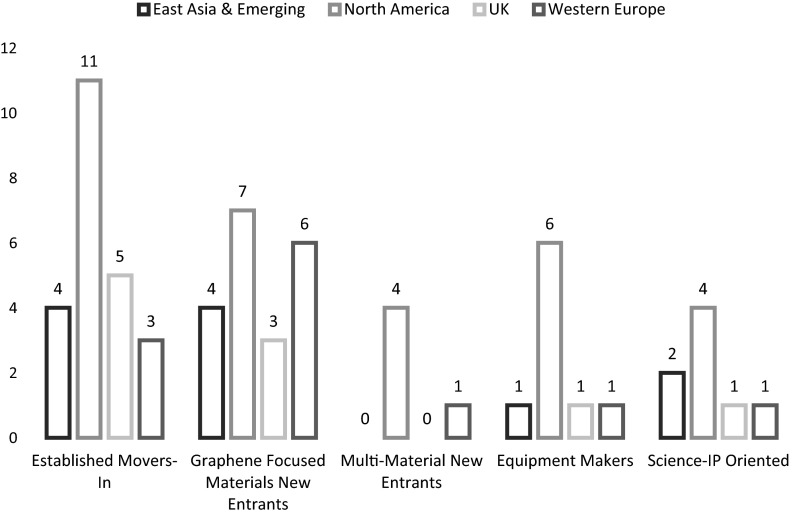
Graphene SMEs clusters by geographical location. *Source* Cluster analysis of 65 graphene small and medium-sized enterprises (SMEs) in study data set

We categorized firms according to their existing or potential products in each value chain positions (Table 2). We validated our categorizations by manual review of the enterprise website coupled with use of secondary information where available. The largest category is material producers with 39 firms (60 % of the total) already offering products, and five firms (7.6 % of the total) are planning to introduce. Ten firms (15.3 % of the total) already produce intermediate products, while 16 (24.61 % of the total) are planning to follow. Ten firms (15.3 % of the total) are active in equipment manufacturing, while one firm (1.5 % of the total) has plans to move into this value chain position. Finally, one firm already introduced final products into the market, while another has such plans (1.5 % of the total each). Some of the firms are active in more than one value chain positions, while some others have actual production in one position and plans in another. Those firms who are located in multiple value chain positions are mostly existing material producers moving downstream by diversifying their (existing and planned) product portfolio with intermediate products or equipment (Fig. 6).

**Table 2:** Firm value chain positions. *Source* Web mining analysis of 65 graphene small and medium-sized enterprises (SMEs) in study data set

	Country groups				Total (All Countries)	Percentage of total (%)
	East Asia and emerging	North America	UK	Western Europe		
Material producers						
Active	4	21	4	10	39	60.0
Planning	2	0	1	2	5	7.7
Intermediate producers						
Active	2	6	1	1	10	15.4
Planning	1	9	4	2	16	24.6
Equipment manufacturers						
Active	2	6	1	1	10	15.4
Planning	0	0	1	0	1	1.5
Final product manufacturers						
Active	0	0	1	0	1	1.5
Planning	1	0	0	0	1	1.5

### Cluster analysis

The analysis reported so far organizes the graphene firms in our sample in regional groupings and value chain positions. There are of course other ways to analyze the web-mined data that we have assembled. One approach is to treat each company as an individual unit, then compare their characteristics and strategies with those of other firms to see where there are similar groupings of these factors. In this approach, similarities and differences among the characteristics of the firms are used to construct groups, rather than using a predetermined group typology. The statistical method used is two-step cluster analysis—this allows us to distinguish archetypes of graphene firms based on key variables. We use the SPSS statistical package to conduct the cluster analysis. Two-step cluster analysis allows for clustering on the basis of both scale and categorical variables. On the basis of the Akaike’s information criterion (AIC) statistics, we selected a five-cluster model. This solution resulted in a “silhouette measure of cohesion and separation” of 0.25 which is generally considered as within the acceptable range. The largest cluster comprises 23 firms, with five firms in the smallest cluster (See Appendix Fig. 7).

We analyzed the typical (average) characteristics of these five clusters. These cluster groups represent sets of companies with broad within-group similarities in characteristics and strategies, and broad between-group dissimilarities with the other groups. The five clusters are:

- *Cluster 1* Established Movers-In (23 firms). Most of these firms were founded before 2000. They are not necessarily only based on graphene, but most of these companies have graphene materials and intermediate products in their current and prospective product portfolio. All of the limited numbers of companies that produce final products also belong to this cluster. Therefore, they are spread over the value chain. These companies have very good access to finance. They are also competent in marketing, especially through social media. All of the graphene-related scientific publications were produced by these firms. This cluster includes half of the UK companies.
- *Cluster 2* Graphene-Focused New Entrants: Materials (20 firms). Almost all of these companies are active in producing graphene material. Their linkages to universities, financial markets, and government are relatively lower.
- *Cluster 3* Multi-Material New Entrants: 2D Materials (5 firms). These firms produce a wide variety of 2D materials including graphene as their mention of 2D materials is greatly higher. They are significantly less R&D active. Their links to other businesses and financial markets are particularly lower, while they mention government in their websites significantly more, most probably due to strict health and safety regulations they need to adhere. They tend to mention production methods for graphene and other 2D materials more than other firms. The websites that these companies maintain are considerably larger than other firms. Most companies in this cluster are based in North America.
- *Cluster 4* Equipment Manufacturers (9 firms). Vast majority of these firms are equipment manufacturers, but some of them are also active in producing graphene material. They have good links with financial markets. They also often mention their relationships with other firms and universities (probably as their significant customers). Most companies in this cluster are based in North America.
- *Cluster 5* Science-IP-Oriented Firms (8 firms): These firms are very young—most of them were established after 2010. They are very research active and they produced the majority of the graphene patents filed by graphene-based SMEs. They have very high linkages with universities as well as other businesses. They tend not to mention government in their websites, while almost all of them underline the Nobel Prize in 2010. They also highlight the properties of graphene more than other firms. Some of these firms have started producing materials and intermediate products, but most of them have not yet introduced products or revealed their plans for future releases.

For our Graphene SME sample, there are relatively more UK Established Movers-In than for other geographical regions. Western Europe and Asia have relatively more graphene-focused materials new entrants. North American graphene SMEs are distributed across all categories (Fig. 6).

### Factors influencing the introduction of products

Achieving the economic and commercial benefits anticipated for graphene is contingent on producing products that are demanded in the marketplace. Four-fifths (53) of the firms in our sample have introduced a product to the market, while the remainder (12 firms) plan to introduce products. Once a graphene product is available, a variety of factors will affect its subsequent success, including the nature of demand, the ability to scale-up production and to develop distribution channels, manufacturing processes, and quality, the advantages of the new product over incumbent technologies, competition, access to finance, and possibly regulatory factors. We cannot assess the future likelihood of success from enterprise web sites, but we can develop insights about the factors that influence initial product introduction. To do this, we developed a model predicting the factors influencing introducing a product in the market.

We employed a binary logistic regression approach. This approach is appropriate for predicting the influence of various independent factors on a binary-dependent variable where two outcomes are possible. In this case, the two possible outcomes are product introduced or not introduced. Our first model examines influences on introducing products in general. To investigate the factors influencing product introduction in specific value chain positions, we developed three additional models. We break out material production and equipment manufacturing and combine intermediate and final products (Appendix Table 4).

We performed a model selection exercise that examined different combinations of independent variables. On the basis of this procedure, we excluded some variables. It would be expected that larger websites have more mentions of any specific factor. However, since our web variables are already normalized by website sizes, our models control for this. The resulting set of models selected performed optimally in terms of statistical measures such as log likelihood, Cox & Snell R Square, and Nagelkerke R Square. We also investigated various other diagnostic statistics to optimize the model power and to decide if a different modeling approach would be more appropriate. As these statistics did not indicate an increase in the explanatory power of our models, we do not report alternative approaches, such as a probit regression. Our sample size is relatively small. While this does not influence effect size (e.g., correlation coefficients), it does influence measures of statistical significance.

Our results suggest that, in general, access to finance and the firms’ location are significant factors that are associated with graphene product introductions. Graphene SMEs that report access to financial sources, including venture and equity capital, have a higher probability of reporting products currently available. The attraction of external finance may signal firms that have more promising applications, and firms may benefit from the review, guidance, and credibility that is associated with external finance. Graphene SMEs located in the UK also had a more significant likelihood of product introduction in general, although for the upstream graphene materials location in Asia was also significant. Mentioning other 2D materials turned out to be a significantly negative predictor of introducing a product into the market. In other words, focusing on graphene was more likely to be associated with a product introduction—perhaps because other 2D materials are as yet further away from being ready for the market or because focusing on multiple materials in a resource-constrained SME might diffuse or slow down commercialization capabilities. We also found that patents and scientific publications were not statistically significant predictors of product development in our sample of graphene SMEs. In terms of individual value chain positions, being in the material production value chain position was positively related with being located in East Asia and emerging economies. Furthermore, firm age was significantly related to being active in final and intermediate products production.

A number of web-based variables including linkages with other businesses, government, and universities are also not significant predictors of introducing a product into the market. There might be two effects working here separately or in combination. First, it might be the case that linkages are in fact not good predictors. Second, it might be that for some firms linkages do matter, but they do not publicize these prominently on their websites.

In terms of individual value chain positions, introducing a product in the material production value chain position is positively related with being located in East Asia and emerging economies. Government links are also relatively important for material producers (statistical significance just below the 90 % threshold level). Access to finance is a critical factor for product introductions by equipment makers, while mention of any other 2D materials is a significant and negative predictor for this group of firms. Finally, firm age is significantly related to introducing products in the intermediate and final part of the value chain. Again, graphene patents and publications are not shown as statistically significantly linked with product introductions in any of the value chain positions (Table 3. For detailed reporting of results, see Appendix Table 4).

**Table 3:** Graphene SMEs: factors influencing product introductions. *Source* Web mining analysis of 65 graphene small and medium-sized enterprises (SMEs) in study data set. Results of a binary logistic regression where columns are dependent variables and rows are independent variables (predictors). Cells show the signs of the corresponding coefficient. Significance levels of 90 % or over are denoted with a star (*). For detailed reporting of results, see Appendix Table 5

Factors	Active (in any value chain position)	Active in material production	Active in equipment manufacturing	Active in intermediate or final products
Graphene publications	Negative	Positive	Negative	Positive
Graphene patents	Positive	Positive	Positive	Negative
Age	Negative	Negative	Negative	Positive*
Finance	Positive*	Negative	Positive*	Positive
Business links	Negative	Negative	Negative	Negative
Government links	Positive	Positive	Negative	Negative
University links	Negative	Negative	Negative	Positive
Total graphene properties mentioned	Negative	Positive	Negative	Positive
Total graphene production methods mentioned	Positive	Positive	Negative	Negative
Any other 2D materials mentioned	Negative*	Negative	Negative*	Negative
Location: North America	Positive	Positive	Negative	Negative
Location: UK	Positive*	Positive	Positive	Negative
Location: Western Europe	Positive	Negative	Positive	Negative
Location: East Asia and emerging economies	Positive	Positive*	Negative	Negative

## Discussion and conclusions

Our review of current developments in graphene research and commercialization presents a broad picture of growth and advancement. There has been widespread and worldwide recognition of the original path-breaking UK research which first isolated graphene. In recent years, there has been a massive expansion in scientific research and advancements in understanding graphene and its properties. This has generated further research on related two-dimensional materials. There has also been great interest in acquiring intellectual property protection across a wide range of potential graphene processes and applications, as demonstrated by the expansion of patent applications. Universities, public research institutions, and companies in Europe, Asia, and the USA, as well as in other countries, are active in graphene research and commercialization. A growing number of companies have entered the graphene domain, with some early products already on the market. Policy initiatives and programs to stimulate graphene research and commercialization have been launched in multiple countries.

Yet, at the same time, there is also uncertainty about the trajectory and character of graphene commercialization. Market expectations and forecasts vary. Manufacturing and industrial-scale up are current areas of concern—and while there is an expectation that these can be resolved with further research, development, investment, and experience, the performance-price competitiveness of graphene compared with other incumbent (and new) materials remains a longer-term issue. The significance of potential barriers to commercialization, whether graphene-specific, such as intellectual property thickets and regulatory developments or broader issues such as access to finance, is also undefined. While there is an emergent graphene value chain, we have yet to see how this will develop and, in particular, what will be the relative roles of large incumbent firms and new small start-up companies in this process. To date, relatively few products enabled by graphene are available in the market and these products mostly offer incremental improvements over existing technologies. The process of developing not only more graphene-enabled devices and products, but also more transformational outputs, is still at an early stage. Such products will need not only to be producible at scale, but also commercially viable, with a premium of price and/or performance over incumbents, and able to gain market acceptability (without significant environmental health and safety concerns).

Uncertainty and ambiguity are, of course, inherent in the process of technological development, especially for novel and disruptive technologies. Moreover, as the path-breaking nature of the underlying research on graphene and its novel properties is acknowledged, it is perhaps inevitable that there will be hype and over-expectation as to the scale and scope of potential commercial applications. The use of hype to boost an emerging technology is evident in elsewhere in nanotechnology and in other emerging technologies (Meyer 2007; van Lente et al. 2013), and there have been calls to better validate such claims (Dedehayir and Steinert 2016). Enterprise web mining offers an avenue to track downstream applications, and our work has identified the technologies and products that graphene SMEs are targeting. While further effort to develop new intelligence and foresight will not eliminate uncertainty, it can inform deliberation and decision-making by providing evidence and insight on developmental pathway, key actors and drivers, and potential outcomes. Updated and validated information on trajectories and developments in innovation in new technologies is vital today for business, researchers and research managers, sponsors and funders, and policymakers. In the world of innovation, sources of information about business and commercialization strategies are often fragmented. Surveys of businesses have inherent time lags and may not be available on a comparative cross-country basis, while proprietary studies are often selective (and expensive). The analysis of patents is a frequently used method and although helpful also has well-known limitations, including measuring invention rather than process or downstream product innovation. Our analysis emphasizes this point. For example, we see that UK companies are submitting fewer graphene patent applications than competitors elsewhere, especially in Asia and the USA. Nonetheless, our analysis of graphene SMEs suggests that the UK is developing a cohort of graphene-oriented SMEs engaged in graphene research and innovation. Although currently focused on producing advanced graphene materials, the UK cohort is signaling plans to develop more intermediate graphene products—which should have higher value in the marketplace. More broadly, our findings suggest that policy consideration needs to go beyond university R&D and concerns with patenting (important as both are) to ensure attention to the introduction and scale-up of downstream intermediate and final graphene products and associated support for market identification. Technology intermediary organizations are likely to be important in these next stages of graphene development. However, also likely to be important are further cross-cutting measures to enhance public and private financial support for high-technology firms, including graphene SMEs, and to build strong business, university, and government relationships that encourage and expand commercialization. Attention might be given to encouraging more existing technology-oriented SMEs to consider how graphene might enhance their applications, in addition to measures to encourage further dedicated new graphene start-ups and university spin-outs. Enhancing linkages and networks between graphene-oriented SMEs and larger firms might also be productive.

In this paper, we have further explored a promising new approach to provide intelligence and insight for the analysis of business developments and innovation in emerging technological fields—through mining information from unstructured online sources such as enterprise web pages (also known as web mining). This approach is appropriate for emerging technologies that have developed in conjunction with the widespread deployment of web pages by companies, for example synthetic biology. Available structured sources are not comprehensive in capturing the innovation strategies of technology-oriented companies, especially smaller firms who may not patent extensively and who are often privately owned. Yet, increasing amounts of information about such firms are available through unstructured online sources. These data are more readily extracted for SMEs than for larger companies. SMEs are often vital in pioneering new technological innovations in emerging sectors. The online information that is publicly and openly reported by SMEs can be further combined with established structured databases including data on patenting, industry reports, and case studies to facilitate real-time and ongoing monitoring, mapping, and analysis.

However, there are limitations. There are difficulties in applying the web content analysis method to large multi-nationals (and their large and complex web sites). The interpretation of what is reported on enterprise web sites is suggestive but can benefit from further validation using other methods. In the current study, we are also limited by the target enterprise population size. We have focused on an emerging technology domain that is of great interest and we have captured detailed data on more graphene SMEs than prior studies. However, at this stage of development, the number of SMEs that are actively engaged in graphene development is relatively small. In further development of enterprise content analysis, it would be important to target domains where there are thousands, if not tens of thousands, of enterprises. This would require requisite scale-up in data analytic capabilities. In this study, we were able to capture the presence of enterprise business and technology terms; in future work, opportunities should be explored to use more sophisticated content analytic methods that can capture topic context as well as the presence to improve interpretation of meaning and significance. Improved capabilities to analyze non-English enterprise websites are also needed. In short, enterprise web content analysis is still exploratory and has limitations as well as advantages. Nonetheless, this approach adds a useful further and new dimension to the information sources available to track and monitor developments in new technologies. While we have discussed the case of graphene companies, it is an approach that can be applied to other areas of emerging technology as part of a larger process of exploring what can (and also what cannot) be additionally learned for innovation management and policy from text mining and data analytic approaches using the expanding array of online sources that now are available.

## Figures and Tables

**Fig. 7:**
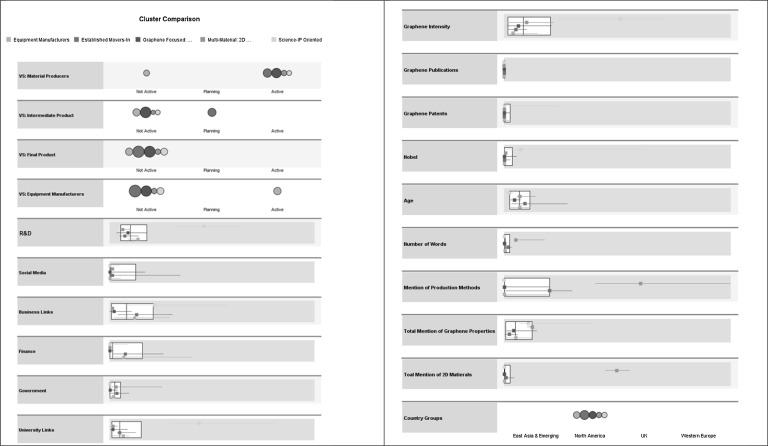
Characteristics of the five graphene SME clusters. *Source* SPSS twostep cluster analysis based on web mining analysis of 65 graphene small and medium-sized enterprises (SMEs) in study data set

## Appendix

See Tables 4 and 5; Fig. 7.

**Table 4:** Text mining rules

Topic	Research question	Notes	Rules
Characteristics of enterprise	Company name URL(s)		Manual
Year of establishment	When was the company established?		Manual
Location	Where is the company located? Which countries does the company operate in?		Manual
Lines of business	What are the company’s main lines of business?	E.g., manufacturing, services	Rule 1: (manufactur* OR produc*)
			Rule 2: (consult* OR |servic*)
			Manually clean
Graphene targets and value-stream position	What graphene products or applications does the company offer (e.g., products preceded by graphene or with nearby mention of graphene)?	There are four categories:	Rule 1: (GNP OR graphene* dispersion OR graphene* powder OR nano* platelets) NEARBY (develop* OR introduce* OR *manuf* OR produc* OR provide*)
		1. Material Producers: they produce the	
		2. Material or flake	
		3. Intermediate products	
		4. Equipment	
		5. Graphene-enabled final products	
			Rule 2: (functional* OR ink* OR master*batch*) NEARBY (develop* OR introduce* OR manuf* OR produc* OR provide*)
			Rule 3: (equipment* OR tool* OR CVD) NEARBY (develop* OR introduce* OR manuf* OR produc* OR provide*)
			Rule 4: (consumer*) NEARBY (develop* OR introduce* OR manuf* OR produc* OR provide*)
			Manually clean
Graphene functionality	What functional characteristics of graphene are highlighted	Graphene NEAR faster, quicker, stronger, thermal, stiffness, elasticity, flexibility, conductivity, transparency, permeability, protective, barrier, etc.	Extract nearby word to “graphene”
			List clean-up (with stemming) adjectives
		see http://www.graphene.manchester.ac.uk/story/properties/	
Graphene production method	What graphene production method is being used?	CVD, chemical vapor deposition, SiC, sicon carbide synthesis, exfoliation, mechanical exfoliation, liquid-phase exfoliation, molecular assembly	Rule: keywords:
			Epitax*
			Exfoliation
			Intercal*
			Molecular assembly*
			Reductio*
			Unzip*
			Deposition
			CVD
			Nanotube*
			Manually clean
		See here for production methods (and also properties/functionality)	
		http://www.nanowerk.com/spotlight/spotid=34184.php	
Other 2-D materials	What 2-D materials does the company offer	(2D; two-dimensional; atomic-scale thickness; atomically thin crystals) AND/OR (boron nitride, hBn, h-BN; transition metal dichalcogenides, TMD; complex oxides) AND NOT graphene	Rule: (boron nitride OR oxide OR Germanane OR h-BN OR HBN OR hBn OR MDS OR metal dichalcogenides OR Molybdenum disulfide OR MoS2 OR Silicene OR TMD)
		See a list non-comprehensive list here: http://pubs.acs.org/doi/full/10.1021/nn400280c	
			Manually clean
Graphene intensity	What is the “graphene intensity” of the company’s products or applications?	(Mentions of graphene in products)/(all mentions of products)	Rule: count number of times graphene appears/total words
Research and development	Does the company undertake research (R&D)? Are there products or applications under development, if yes what products and applications?		Rule: (development*activity OR development*cent* OR development*cycle OR development*efforts OR development*facility* OR development*phase OR development*process* OR development*program* OR development*project* OR development*research OR lab* OR product*development* OR R&D OR research* OR research& OR *development OR Research*development OR RnD OR science* OR scientist* OR technical*development* OR technological*development* OR technology*development*)
			Manually clean
Markets	How does the company market its products?		Rule 1 = ($* OR *shop* OR £* OR €* NOT (workshop*))
			Manually clean
Government linkages	What governmental support has been provided to the company? (Government grants, subsidies, participation in government or quasi-governmental programs)		Rule: policy* OR policies OR government* OR regulate*
			Manually clean
Business linkages	What other businesses does the enterprise link with and what forms are those linkages?	E.g., joint ventures; partnerships; supply chain linkages; discussion of relationships with customers	Rule: (agreement OR alliance* OR association* OR joint*venture* OR partner* OR co*operation*)
			Manually clean
University linkages	What are the enterprise’s links with universities and colleges? And what forms do these links take?		Rule: university*
			Manually clean
Finance	What sources of private sector finance are highlighted?		Capital*
			Equity*
			Funding*
			Venture*capital*
			Manually clean
Nobel	Do they mention the Nobel Prize (related to graphene)?		Rule: (geim* OR novoselov* OR nobel prize*)
			Manually clean
Social media	What social media methods are used?	Twitter; Facebook; Linked-in; Other (Google Plus, YouTube, Vimeo, Flickr)	Rule: (blog* OR *facebook* OR linked*in* OR twitter* OR youtube*)
			Manually clean

**Table 5:** Detailed results of the regression analysis. *Source* Web mining analysis of 65 graphene small and medium-sized enterprises (SMEs) in study data set. Results of a binary logistics regression where columns are dependent variables and rows are independent variables (predictors). Significance levels of 90 % or over are denoted with a star (*). “−2 Log likelihood”, “Cox & Snell R Square,” and “Nagelkerke R Square” are model level statistics used to compare different models

Factors	Active (in any value chain position)		Active in material production		Active in equipment manufacturing		Active in intermediate or final products	
	Coefficient	Significance	Coefficient	Significance	Coefficient	Significance	Coefficient	Significance
Graphene publications	−0.11635	0.287366	0.054547	0.639095	−4.35172	0.998379	0.057108	0.566747
Graphene patents	0.013479	0.798586	0.024677	0.627027	0.022442	0.864103	−0.05012	0.679687
Age	−0.03469	0.347588	−0.04678	0.196897	−0.01319	0.795339	0.0941*	0.064259
Finance	1.81117*	0.043849	−0.28894	0.697025	3.984863*	0.038744	1.231949	0.413448
Business links	−1.37018	0.227652	−0.05308	0.953101	−1.75812	0.222243	−1.14297	0.537043
Government links	0.300406	0.734458	0.940486	0.184387	−1.34136	0.197128	−0.04797	0.967054
University links	−1.67677	0.241207	−1.17146	0.246573	−2.55621	0.131521	17.75487	0.998479
Total mention of graphene properties	−1.01875	0.458156	0.737063	0.509839	−3.90754	0.344891	0.013377	0.993229
Total mention of graphene production methods	2.189844	0.44879	1.655503	0.349662	−0.9856	0.653659	−0.88566	0.678531
Mentioned any other 2D material	−1.88155*	0.099055	−0.18174	0.842117	−2.95464*	0.092068	−23.4164	0.997405
Location: North America	0.950636	0.376449	0.010642	0.98849	−0.59907	0.505091	−6.29549	0.998382
Location: UK	2.044407*	0.091096	0.623899	0.46505	1.438128	0.267727	−6.42595	0.998348
Location: Western Europe	0.409422	0.660353	−0.99637	0.205313	0.081107	0.952555	−6.38837	0.998358
Location: East Asia and emerging	1.421985	0.171341	1.813318*	0.039297	−0.30678	0.80863	−7.4843	0.998076
Number of observations	65		65		65		65	
−2 Log likelihood	52.03		72.58		37.696		39.628	
Cox & Snell R^2^	0.44336		0.236372		0.553521		0.540048	
Nagelkerke R^2^	0.591147		0.315163		0.738027		0.720064	
